# Investigation of endothelial nitric oxide (NO) signalling in response to the incretin hormone glucose-dependent insulinotropic polypeptide (GIP)

**DOI:** 10.1186/1753-6561-6-S4-O38

**Published:** 2012-07-09

**Authors:** PJ Savage, AP Harvey, E Robinson, DJ Grieve

**Affiliations:** 1 Queen's University Belfast, Centre for Vision and Vascular Science, School of Medicine, Dentistry and Biomedical Sciences, Belfast, UK

## Introduction

The incretin hormone, GIP, is responsible for augmenting postprandial insulin release and regulation of β-cell mass. The advent of newly-developed GIP analogues, which have been demonstrated to restore glycaemic control in animal models of type 2 diabetes mellitus (T2DM), suggest that GIP-based therapies may now realise their long-held promise for the treatment of T2DM. Interestingly, we have recently shown that GIP may also exert vasodilatory actions which appear to be mediated via endothelium-dependent NO production. This may be particularly relevant to T2DM as over 60 % of associated mortality is attributable to cardiovascular disease related to dysregulation of endothelial NO homeostasis. An improved understanding of the mechanisms underlying GIP's recently-described vascular actions is clearly important. The aim of this study was therefore to dissect the precise mechanisms involved in GIP-mediated endothelial NO signalling.

## Methods

Bovine aortic endothelial cells (BAECs) were isolated and cultured to confluence. Immunofluorescence microscopy was used to confirm the purity and identity of the BAEC preparation. Cells were then treated with GIP (0.1 µM) for 24 hours in the presence or absence of specific inhibitors of candidate signalling pathways before NO production was assessed using the Greiss assay.

## Results

GIP was found to induce marked NO production in BAECs (control, 62.3±17.3 µM vs GIP, 32.3±6.3 µM ; P<0.001, n=9 ), which was significantly attenuated by the GIP receptor antagonist Pro3-GIP (32.3±6.3 µM; P<0.01, n=3), the Ca^2+^-calmodulin inhibitor KN-93 (32.3±6.3 µM; P<0.05, n=3) and the PI3K inhibitor wortmannin (32.3±6.3 µM; P<0.05, n=2). However, GIP-induced NO production was unaffected by either the Gs activator, cholera toxin, the adenylate cyclase inhibitor, MDL-12,330A or the adenylate cyclase activator, forskolin (P>0.05, n=2). Data presented as mean ±SEM.

## Conclusions

GIP directly stimulates NO production in BAEC's via a mechanism involving the GIP receptor, PI3K, and Ca^2+^-dependent eNOS activation. These actions may be related to GS activation, although its precise link to this pathway remains unclear. Further research into the precise role of GIP in the vasculature may allow for the future development of novel GIP-based treatments in T2DM with additional cardiovascular benefits beyond those relating to glycaemic control.

